# Construction of a Transparent, Robust, Shape-Memory and Self-Healing MDI-Based Polyurethane Elastomer

**DOI:** 10.3390/polym17091243

**Published:** 2025-05-02

**Authors:** Haichun Dang, Ziliang Zhang, Ruibing Sun, Yunlun Li, Mengyu Lin, Siting Yang, Maoyong He, Zhaozan Xu, Xiangcheng Bian

**Affiliations:** 1 Department of Materials Engineering, Taiyuan Institute of Technology, Taiyuan 030008, China; danghc@tit.edu.cn (H.D.); 2220700125@tit.edu.cn (Z.Z.); 2120700702@tit.edu.cn (R.S.); 2120700813@tit.edu.cn (Y.L.); 2220700618@tit.edu.cn (M.L.); 2220700628@tit.edu.cn (S.Y.); hemy@tit.edu.cn (M.H.); 2 Institute of Resources and Environmental Engineering, Shanxi University, Taiyuan 030006, China

**Keywords:** transparency, robust, self-healing, shape-memory, polyurethane elastomer

## Abstract

Integrating strong mechanical properties and excellent optical properties for self-healing materials is challenging in both academia and industry. Robust self-healing polyurethane elastomers are expected to have superior mechanical properties, transparency, remarkable healing capability, and shape-memory performance via the adjustment of chemical and microphase separation structure. Herein, a robust transparent self-healable 4,4′-diphenylmethane diisocyanate (MDI)-based polyurethane elastomer containing disulfide bonds and branched structure (MPUE-SS) was synthesized. The chemical and topological structures, compatibility of soft–hard phases, and hard domain size of polyurethane could be adjusted via branched structure and mixed chain extender containing disulfide bonds and 1,4-butanediol (BDO), leading to enhanced self-healing, transparency, and mechanical properties. MPUE-SS exhibited a maximal tensile strength of 40 MPa. The microphase separation structure and reduced crystallinity led to a high transparency of about 91%, close to that of alicyclic polyurethane elastomers. After cutting in half and splicing, the MPUE-SS film recovered more than 95% of the original mechanical properties in 24 h. The shape recovery ratio at 40 °C and shape fixity ratio at −20 °C of MPUE-SS were 96.0% and 99.6%, respectively, higher than those of MPUE without disulfide bonds. Therefore, the chemical, topological structures, and microphase separation of polyurethane could be adjusted to achieve desired self-healing, transparency, shape-memory, and mechanical properties.

## 1. Introduction

The self-healing and shape-memory polymers have received wide attentions due to their advantages, such as self-regulation, damage repair, and lifetime dilation [1,2,3]. They are expected to solve the potential problems facing many high-tech fields, such as textiles, biomedicine, electronics, machinery, and aerospace [4,5]. Among them, polyurethane elastomers (PUEs), usually synthesized from polyols, isocyanates, and chain extenders, have been utilized in various fields owing to their high flexibility, robust mechanical properties, resistance to organic solvents, and outstanding water resistance [6,7]. The shape-memory property of PUEs depends on the microphase separation between soft and hard segments. The temporary shape fixing of PUEs depends on the reversible phase of soft segments, while the shape recovery depends on physical crosslinking associated with hydrogen bonding, crystallization, and polar interactions in the hard segments. The commonly used shape-memory polyurethanes are linear polymers, and they lose the ability to retain and recover their shape after several thermomechanical treatments. In our previous work, a star-shaped shape-memory thermoplastic PUE (STPUE), via adding poly(propylene glycerol) (PPG-3), was developed to enhance the shape-memory and mechanical properties [8]. A highly branched structure was found able to reduce the microphase separation and improve the phase compatibility of STPUE, thereby enhancing its mechanical properties. Recently, dynamic chemistry has been successfully applied in designing self-healing and shape-memory polymers [9,10,11,12]. The disulfide bond, as a reversible dynamic bond, was introduced into the polymer chains to ensure the self-healing of polymer [13,14].

In addition to self-healing, the high transparency, shape-memory, and mechanical properties of thermoplastic PUEs are often required in various applications, such as bulletproof laminated glass, transparent protective products, precision optical lenses, transparent protective coatings, decorative materials, flexible display screens, etc. [15,16,17]. The transparency of PUEs, along with the self-healing and mechanical properties, can be finely tuned by modifying the chemical compositions and molecular weights of hard and soft segments. Recent studies have reported the fabrication of highly transparent and self-healing PUEs using aliphatic or alicyclic isocyanates and disulfide compounds [18,19,20,21]. Kim et al. developed a transparent thermoplastic PUE with super toughness and rapid self-healing, with the toughness and maximum tensile strength reaching 26.9 MJ/m^3^ and 6.8 MPa, respectively, and the self-healing efficiency reaching 75% at room temperature in 2 h [18]. Wang et al. prepared a transparent and colorless PUE using aliphatic disulfide (bis(2-hydroxyethyl)disulfide, HEDS) and aliphatic isocyanate (m-xylylene diisocyanate, XDI) as the hard segment and polytetramethylene ether glycol as the soft segment, with the elongation at break ranging from 825 to 1320% and tensile stress ranging from 3.51 to 8.12 MPa [19]. Therefore, the polyurethane synthesized from aliphatic or alicyclic isocyanates showed good transparency but poor tensile strength owing to the low bond energy of dynamic disulfide bonds. Lai et al. prepared transparent and colorless self-healing PUEs using aliphatic disulfides as the chain extender, which exhibits a fracture strain of over 1600% and a maximal tensile stress of 25 MPa [20]. These studies suggested that the introduction of isocyanates and disulfide compounds can balance the transparency, self-healing performance, and mechanical properties of PUEs.

Though aromatic diisocyanate or disulfide compounds can improve the self-healing and mechanical properties of PUEs, they often lead to reduced transparency due to microphase separation and crystallization in the hard-segment phase. Hard segments used for segmented thermoplastic polyurethanes (TPUs) are usually derived from 1,4-butanediol (BDO) and 4,4′-diphenylmethane diisocyanate (MDI), though there are a few reports on MDI-based PUEs with high transparency, strength, and flexibility. 1,6-Hexanediol can also be used as the chain extender to improve the transparency of segmented polyurethanes due to the low crystallinity in hard segments [22]. To gain the expected high transparency and tensile strength of TPUs, polyurethanes can be synthesized with high contents of hard segments [23,24,25]. However, polyurethanes are too brittle for use in foldable transparent devices (such as goggles, gas masks, etc.) and shape-memory transparent materials. Therefore, the combination of multiple chain extenders is used to decrease the crystallinity and enhance the miscibility between hard and soft and hard segments [26]. Taken together, producing TPU films with high self-healing ability, strong mechanical properties, and high transparency is challenging.

Herein, we constructed a new self-healing MDI-based PUE with disulfide bonds (MPUE-SS), which exhibited superior transparency (close to the aliphatic or alicyclic isocyanates-based PUEs), a maximum tensile strength of 40 MPa, and high shape-memory and self-healing performance. In MPUE-SS, the macromolecular polyols of PPG-3 and poly(ε-caprolactone) (PCL) were adopted as the core and soft segments, respectively. 2,2-Hydroxyethyl disulfide (HEDS) and BDO were utilized as the combined chain extenders to reduce crystallization and enhance microphase separation. By acting as covalent dynamic bonds, disulfide bonds of HEDS could endow the materials with self-healing properties through disulfide exchange [18,20,27,28,29]. The branched architecture of PPG-3 could endow MDI-based polyurethane with high phase compatibility, mechanical properties, and shape-memory performance. Meanwhile, the integration of disulfide bonds could not only facilitate dynamic switching and increase the self-healing performance of PUEs but also control the size of hard segments within a small range.

## 2. Materials and Methods

### 2.1. Materials and Reagents

PPG-3 (Mn = 300, f = 3) was obtained from Shandong Bluestar Dongda (Zibo, China). Poly(ε-caprolactone) (PCL, Mn = 1000, f = 2) was purchased from Sigma-Aldrich (Saint Louis, MO, USA). 1,4-Butanediol (BDO) was obtained from Sinopharm Chemical Reagent (Shanghai, China). 4,4′-Diphenylmethane diisocyanate (MDI) was purchased from Wanhua Chemical Group (Yantai, China). Bis(2-hydroxyethyl)disulfide (HEDS) was obtained from Alfa Aesar (Shanghai, China). All reagents were dried before use.

### 2.2. Synthesis of MPUE and MPUE-SSs

**Scheme 1:**
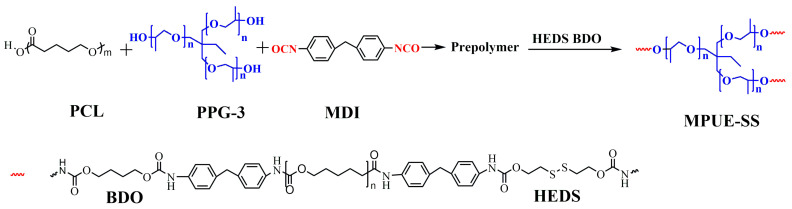
Synthesis of MPUE-SS.

As shown in Scheme 1, MPUE was synthesized using the pre-polymerization method, and then MPUE-SSs with different ratios of BDO and HEDS were fabricated at the corresponding feed ratios. As shown in Table 1, samples of MPUE, MPUE-SS-1, MPUE-SS-2, and MPUE-SS-3 were fabricated with the same molar ratio of OH in diol, NCO in MDI, and OH in extender, but different extender molar ratios of BDO and HEDS in consideration of the effect of HEDS on the self-healing property. Samples of MPUE-SS-4 and MPUE-SS-5 were fabricated with the same extender molar ratios of BDO and HEDS but different molar ratios of OH in diol, NCO in MDI, and OH in extender for adjusting the content of the hard segment. The amount of hydroxyl groups in PPG-3 is 3% of the total hydroxyl groups in PPG-3 and PCL [8]. Briefly, PCL and PPG-3 were vacuum-dried in a three-necked round-bottom flask (500 mL) at 80 °C for 2 h, followed by addition of MDI. The pre-polymerization was conducted with mechanical stirring at 80 °C for 2 h. The pre-polymers were kept at 85 °C for 30 min and defoamed in a planetary vacuum mixer for 3 min. BDO and HEDS were then added dropwise at 90 °C, followed by chain extension at 90 °C for 2 min with stirring. After pouring into a mold at 120 °C, the viscous mixture was pressed under 150 bar for 40 min. After curing at 100 °C for 20 h, the as-prepared films were maintained at room temperature for 7 d.

### 2.3. Characterization of Structural and Physicochemical Properties

The reaction between hydroxyl groups and isocyanate was characterized by using FT-IR spectroscopy (TENSOR 27 FT-IR, Bruker Optik GmbH, Ettlingen, Germany). The presence of disulfide bonds in MPUE-SSs was detected using Raman spectroscopy on an Invia-Qontor Raman spectrometer (Renishaw, London, UK) at 532 nm in the range 3200–400 cm^−1^. The composition of MPUE and MPUE-SSs was determined using ^1^H-NMR spectroscopy on a Bruker AVANCE III 400 MHz spectrometer. DMSO-d6 was used as the solvent. The UV–visible spectra of MPUE and MPUE-SSs were obtained in transmittance mode from 300 to 800 nm. The weight-average molecular weights (*M*_w_), molecular weight distribution, and number-average molecular weights (*M*_n_) of MPUE and MPUE-SSs were measured using gel permeation chromatography (GPC, Malvern, Malvern, UK). Monodisperse polystyrene was used for calibration, and dimethyl formamide was utilized as the mobile phase (0.7 mL/min).

The crystal structure of MPUE and MPUE-SSs was analyzed by using X-ray diffraction (XRD) on a TD 3700 diffractometer (Fangyuan Instruments, Dandong, China) at a scanning rate of 9.6°/min over the range of 10° to 50°. The microphase separation structure was characterized by using small-angle X-ray scattering (SAXS) on a SAXS ess mc^2^ system (Anton Paar, Graz, Austria) at room temperature. The wavelength of Cu K_α1_ irradiation was 1.5406 Å.

Thermal properties of MPUE and MPUE-SSs were analyzed on a Q20 differential scanning calorimeter (DSC, TA Instruments, New Castle, PA, USA). Briefly, after heating from −80 °C to 250 °C at 10 °C/min, the samples were cooled to −80 °C at 10 °C/min under nitrogen purging. Dynamic mechanical properties (loss factor tan δ and storage modulus) were characterized on a Q800 dynamic mechanical analyzer (DMA, TA Instruments, New Castle, PA, USA) in the temperature range of −80 °C to 150 °C at a heating rate of 3 °C/min. After tensile tests, the fracture surface morphology of MPUE and MPUE-SSs was observed using scanning electron microscopy (SEM, JSM-7200F, JEOL, Akishima-shi, Japan).

### 2.4. Characterization of Mechanical Properties

The self-healing performance and tensile strength of MPUE and MPUE-SSs were determined using an AI-7000M universal testing machine (GOTECH, Dongguan, China) at 100 mm/min, according to ASTM D638 (type III). Before testing, samples were pre-conditioned at 23 °C for 24 h under 50% relative humidity. Each sample was tested five times.

To determine the self-healing performance, MPUE-SS films with the same dimensions as above were scratched to about 50% thickness using a razor blade, followed by heating at 120 °C for 24 h. The healing efficiency (η) was calculated by Equation (1).

(1) $$\eta \left(\%\right)=\frac{\sigma_{0}}{\sigma_{h}}\times 100\%$$

where σ_0_ and σ_h_ are the tensile strength of the original and self-healed samples, respectively.

### 2.5. Shape-Memory Property

The dual shape-memory property of MPUE and MPUE-SSs was measured using the cyclic thermal–mechanical analysis on a Q800 dynamic mechanical analyzer. First, the specimen (shape A) was heated to *T*_high_ (*T*_high_ = 40 °C > *T*_g, s_) under the unloading condition and maintained for 10 min. Then, an external force was applied at a certain rate to a certain strain, and the temporary shape I (shape B) was defined as εs1, load. After cooling to *T*_low_ (*T*_low_ = −20 °C), the stress of the specimen was released, and the resulting strain was defined as ε_s1_. Third, the specimen (shape B) was thermally treated at 40 °C. When it recovered to shape A, the strain was recorded as ε_s0,R_. This measurement was repeated three times. The shape fixity ratio (*R*_f_) and shape recovery ratio (*R*_r_) were calculated as follows [8]:

(2) $$R_{f}=\frac{\varepsilon_{S1}-\varepsilon_{0}}{\varepsilon_{S1,load}-\varepsilon_{0}}\times 100\%$$

(3) $$R_{r}=\frac{\varepsilon_{S1}-\varepsilon_{S0,R}}{\varepsilon_{S1}-\varepsilon_{S0}}\times 100\%$$

## 3. Results and Discussion

### 3.1. Structure of MPUE and MPUE-SSs

**Figure 1:**
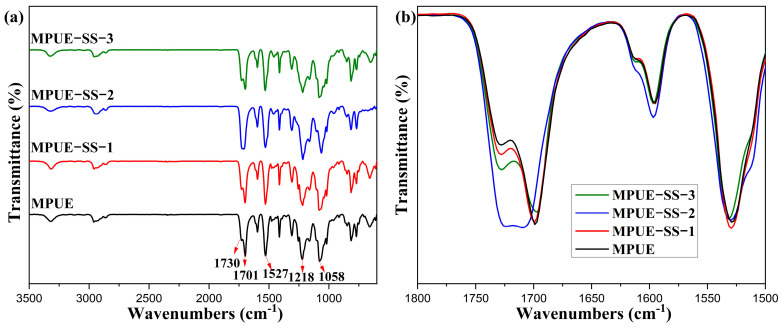
FT-IR spectra in the range of (**a**) 3500–400 cm^−1^ and (**b**) 1800–1500 cm^−1^ of MPUE and MPUE-SSs with different ratios of BDO and HEDS.

The physicochemical properties of PUEs depend on the chemical structure, molecular weights, and physical crosslinking of soft and hard segments. Previously, it has been demonstrated that the addition of PPG-3 could enhance the mechanical properties of PUEs [8]. In the FT-IR spectra of MPUE and MPUE-SSs (Figure 1), the peak at 1701 cm^−1^ was attributed to urethane linkage (ν-NHCO), and the peak of NCO groups in MDI at 2260 cm^−1^ was not found [15], which confirmed the formation of PUEs. The peak at 1595 cm^−1^ corresponded to the skeletal vibration of C=C in the aromatic ring, which was coupled with the peak at 814 cm^−1^ corresponding to the out-of-plane bending of C-H in 1,4-disubstituted benzene. The peak at 1527 cm^−1^ corresponded to the N-H bending vibration, a characteristic peak of polyurethane. The peaks at 1058 and 1218 cm^−1^ corresponded to ether groups (C-O-C). These results confirmed the successful synthesis of MPUE and MPUE-SSs. In addition, the peaks at 1701 and 1730 cm^−1^ corresponded to ordered hydrogen-bonded and free hydrogen-bonded carbamate carbonyl groups, respectively [30]. It is worth noting that MPUE and MPUE-SSs contained a fairly high content (see Table 1 and Table S1) of hard segments. The peak intensity of MPUE at 1701 cm^−1^ was relatively stronger than that at 1730 cm^−1^. In general, the strong hydrogen bonding in hard segments is beneficial for microphase separation. With the addition of HEDS, the peak intensity at 1701 cm^−1^ decreased slightly, while the peak intensity at 1730 cm^−1^ increased slightly. Particularly, the absorption peak intensity of MPUE-SS-2 at 1730 cm^−1^ increased remarkably at the BDO:HEDS ratio of 2:2, indicating a significant increase in the free hydrogen-bonded carbamate carbonyl groups and a relative decrease in the ordered hydrogen-bonded carbamate carbonyl groups of MPUE-SS-2.

**Figure 2:**
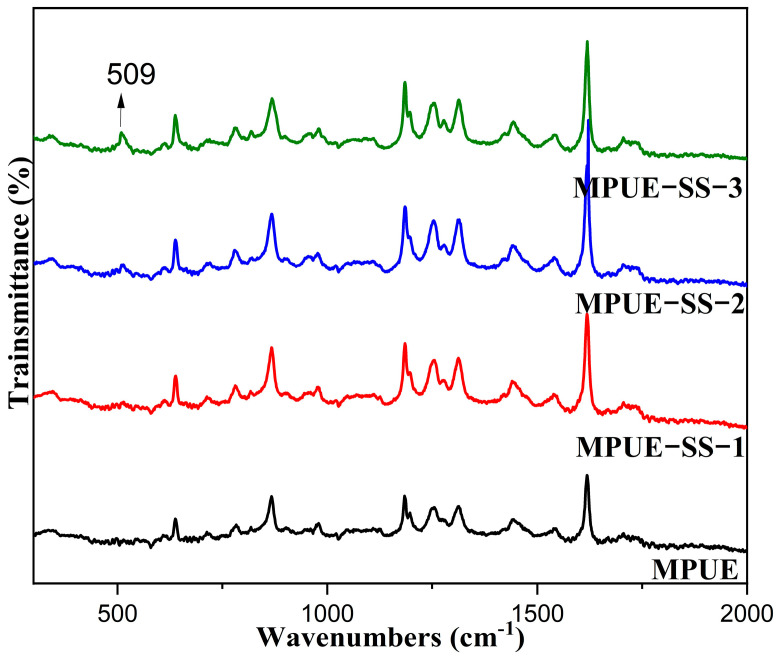
Raman spectra of MPUE and MPUE-SSs with different ratios of BDO and HEDS.

The introduction of disulfide bonds in MPUE-SSs was determined by using Raman spectroscopy (Figure 2). The peak at 509 cm^−1^ was ascribed to the S-S stretching in HEDS [19], indicating that disulfide bonds were successfully incorporated in MPUE-SSs.

**Figure 3:**
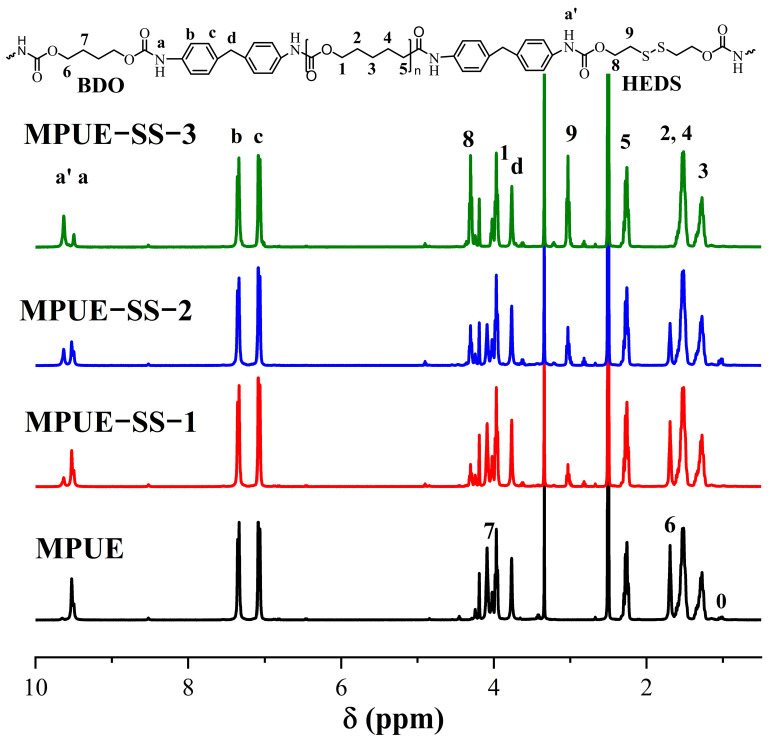
^1^H-NMR spectra of MPUE and MPUE-SSs with different ratios of BDO and HEDS.

Figure 3 shows the ^1^H-NMR spectra of MPUE and MPUE-SSs. The peaks at 7.35 (b) and 7.07 (c) ppm corresponded to the protons in benzene rings [8]. The peaks at 9.52 (a) and 9.63 (a’) ppm corresponded to the N-H of urethane groups in BDO/PCL and HEDS, respectively. The peaks at 1.28 (H-3), 1.51 (H-2,4), 2.29 (H-(5), and 3.97 (H-1) ppm were ascribed to the protons of the PCL chains [31]. The signals H-(7) at 4.10 ppm and H-(6) at 1.69 ppm corresponded to the protons of BDO. The peaks at 4.30 (H-8) and 3.02 (H-9) ppm appeared at the positions of HEDS. The peak at 1.00 ppm (H-0) was attributed to the protons of the -CH_3_ group in PPG-3 [8]. These results confirmed the successful formation of MPUE and MPUE-SSs through polymerization.

GPC was used to determine molecular weights and molecular weight distributions of MPUE and MPUE-SSs (Table 1). There was no significant difference in molecular weights between MPUE and MPUE-SSs when adjusting the content of HEDS in extender with a consistent molar ratio of diol, MDI, and extender. However, the molecular weight and PDI values (Table 1) of MPUE-SSs were increased when increasing the molar ratio of hydroxyl groups in diol to extender from 1:3 to 1:2 or 1:1. This is possibly because the increased diol could enhance the molecular weight of branched pre-polymer.

**Figure 4:**
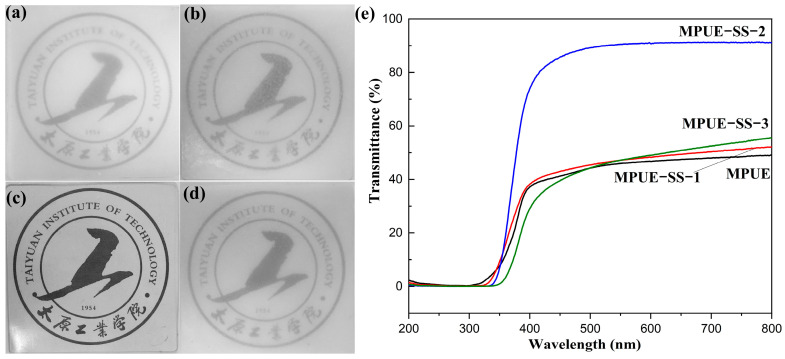
Comparison of the transparency between MPUE and MPUE-SS films covering on our school badge (thickness: 2 mm). (**a**) MPUE, (**b**) MPUE-SS-1, (**c**) MPUE-SS-2, (**d**) MPUE-SS-3, and (**e**) UV–visible transmittance spectra of MPUE and MPUE-SSs with different ratios of BDO and HEDS.

The UV–visible spectra and representative photographs of MPUE and MPUE-SS films are shown in Figure 4. The combined use of HEDS and BDO as the chain extenders could remarkably increase the transparency of MPUE-SSs (Figure 4b–d) to varying degrees compared to that of MPUE (Figure 4a). Particularly, the transmittance of MPUE-SS-2 film was 91% at 550 nm, which was much higher than the 41% of MPUE (Figure 4e), indicating that the optimal ratio of HEDS and BDO was 2:2.

**Figure 5:**
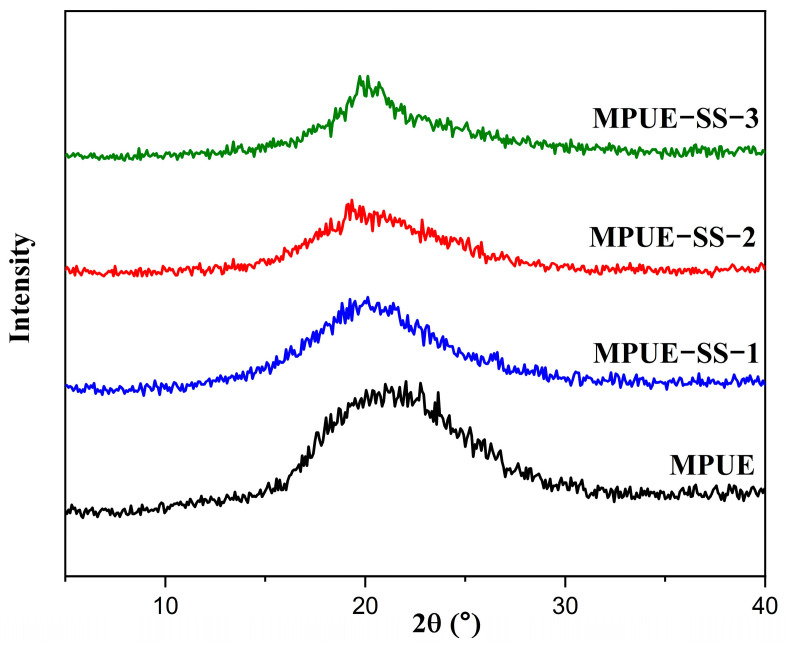
XRD patterns of MPUE and MPUE-SSs with different ratios of BDO and HEDS.

The crystallization of PUEs is an important factor for their mechanical properties. For example, the strength, solvent resistance, and softening point of PUEs increase with increasing degrees of crystallization. However, the transparency, elongation at break, and solubility decrease with increasing degrees of crystallization. Here, XRD was carried out to identify the changes in the crystalline structure of MPUE and MPUE-SSs induced by different ratios of BDO and HEDS (Figure 5). The XRD patterns of PUEs exhibited a sharp characteristic peak of semi-crystalline polyurethane at 2θ = 19.2°. Notably, the peak intensity of MPUE-SS-2 decreased compared to those of MPUE, MPUE-SS-1, and MPUE-SS-3, which could be related to the reduced crystallinity of MPUE-SS-2 at the BDO:HEDS ratio of 2:2 (Table 2) and lead to its high transparency (Figure 5).

**Figure 6:**
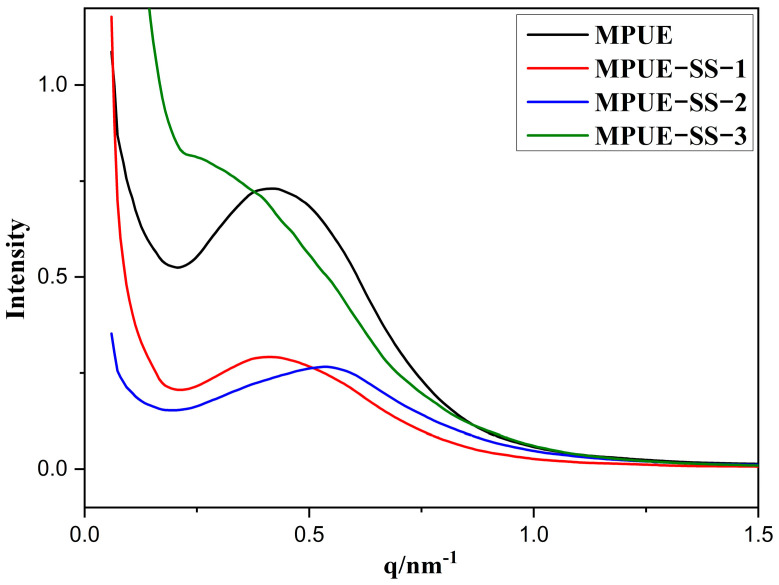
I(q) profiles of MPUE and MPUE-SSs with different ratios of BDO and HEDS.

It was observed whether the addition of HEDS could weaken the microphase separation of PUEs without changing their mechanical properties using SAXS (Figure 6). The interference peak of the scattering vector q indicates the occurrence of microphase separation of the soft and hard segments in PUEs [32]. With increasing contents of HEDS, the intensity of interference peaks of MPUE-SSs decreased gradually (Figure 6), especially for MPUE-SS-2, indicating less aggregation of hard segments and weak microphase separation in MPUE-SSs. The peak position indicates the interdomain distance. Here, the scattering vector q of MPUE-SS-2 shifted toward a higher value (Figure 6), indicating that the interdomain distance of the hard phase decreased at a BDO:HEDS ratio of 2:2. The regularity in domain size was evaluated in terms of the full width at half the maximum of the peak [33]. The full width at half the maximum of the peak in the curves of MPUE-SS-1 and MPUE-SS-2 was greater than that of MPUE and MPUE-SS-3 (Figure 6), suggesting that the BDO:HEDS ratio affected the regularity of the hard domain size. The variations in peak intensity (q ≈ 0.5 nm^−1^) and full width at half the maximum of the peak indicate that the hard domain size was substantially reduced by the combined addition of HEDS and BDO.

**Figure 7:**
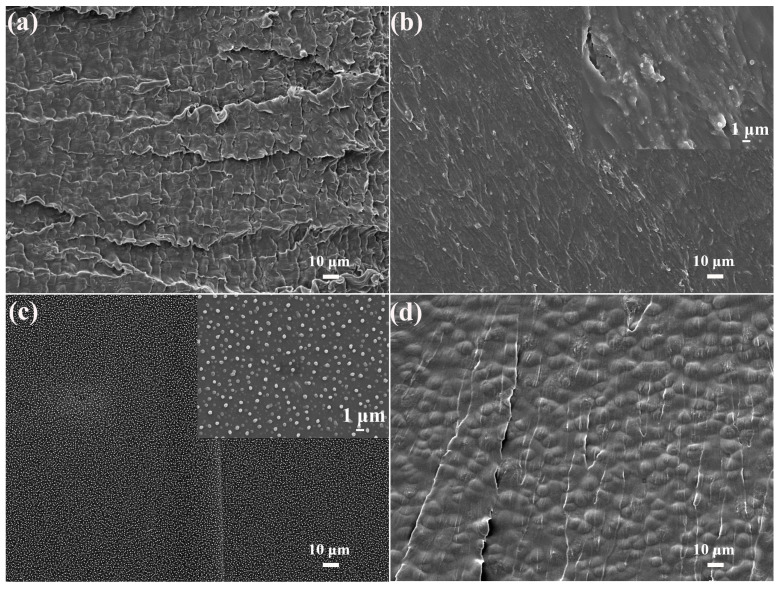
SEM images of tensile fracture surfaces of MPUE and MPUE-SSs with different ratios of BDO and HEDS showing the aggregation of hard segments (bright areas) and soft segments (dark areas). (**a**) MPUE, (**b**) MPUE-SS-1, (**c**) MPUE-SS-2, and (**d**) MPUE-SS-3.

The microphase separation structure of MPUE and MPUE-SSs was further verified by SEM (Figure 7). The SEM images showed that both soft segments (dark areas) and hard segments (bright areas) were aggregated on the fracture surfaces of MPUE and MPUE-SSs. By serving as the matrix of PUEs, the hard segments in the soft phase endowed them with high stretchability [20]. In the SEM images (Figure 7a,d), the spherulites (about 10 μm in diameter) were observed in MPUE and MPUE-SS-3. Similarly, spherulites (1–3 μm in diameter) of hard segments in polyurethane are surrounded by soft segments [8,34,35]. With the combined use of BDO and HEDS as chain extenders, the size of the hard segment domains decreased remarkably (Figure 7b). The hard segment domain sizes in MPUE-SS-1 were about 100–500 nm, and the spherulites were partially agglomerated. As for MPUE-SS-2, however, a uniform dispersion of spherulites with a relatively similar size of 100–500 nm was observed on the fracture surface, without aggregation (Figure 7c).

### 3.2. Thermal Behaviors

**Figure 8:**
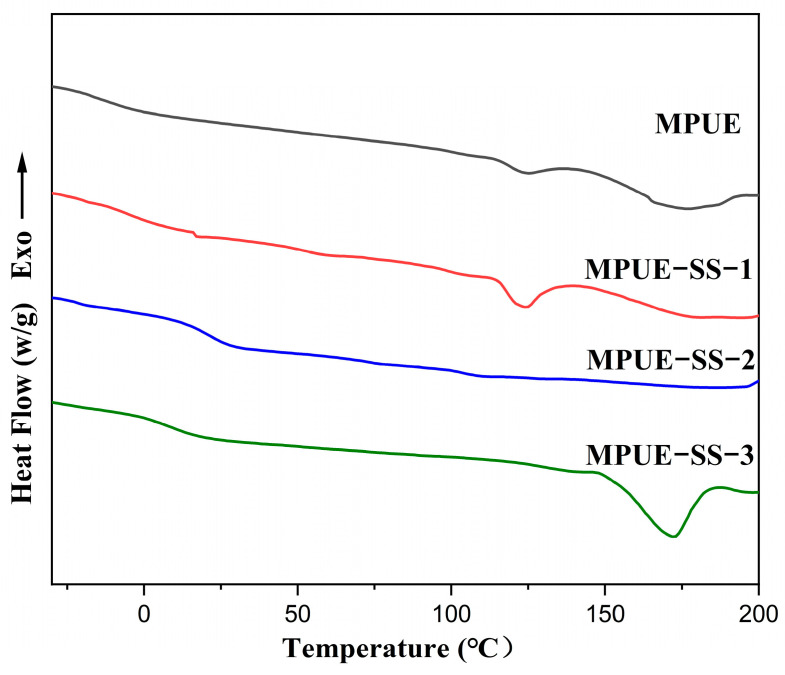
DSC curves of MPUE and MPUE-SSs with different ratios of BDO and HEDS.

**Figure 9:**
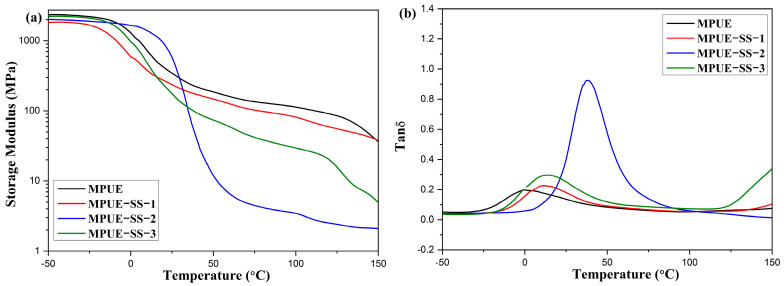
DMA curves of MPUE and MPUE-SSs with different ratios of BDO and HEDS. (**a**) Storage modulus, and (**b**) Tan δ.

As shown in Figure 8, an obvious *T*_g_ of soft segments (*T*_g, s_) and a weak *T*_g_ of hard segments (*T*_g, h_) were observed in MPUE and MPUE-SS, indicating the occurrence of microphase separation of soft and hard segments in PUEs. Two glass transition temperatures (*T*_g, s_ = −15.9 °C, *T*_g, h_ = 48.6 °C) were observed in the thermogram of MPUE (Table 2, Figure 9). With the increasing contents of HEDS, the *T*_g, s_, and *T*_g, h_ of MPUE-SS increased, especially for MPUE-SS-2 (*T*_g, s_ = 20.4 °C, *T*_g, h_ = 71.1 °C). This phenomenon could be explained by the decreased hard domain size and uniform dispersion of hard domains in the soft phase, as shown in Figure 7. These sparsely scattered hard domains could act as a dispersed phase for the soft segment phase to reduce the free volume of the soft segments, leading to an increase in *T*_g, s_. The slightly improved hard segment content of MPUE-SSs could also increase the *T*_g_. In addition, the decrease in the difference between *T*_g, h_ and *T*_g, s_ indicates that the increasing contents of HEDS could weaken the microphase separation of hard and soft segments and improve the compatibility of MPUE-SSs.

Additionally, there were two endothermic thermal behaviors in MPUE at around 110~170 °C (*T*_m1_) and 170~200 °C (*T*_m2_), which were ascribed to the melting of long-range ordered structures and microcrystalline structures, respectively [36,37,38]. There was a melting peak of the microcrystalline structure (*T*_m2_) in the DSC curve of MPUE-SS-3. The melting enthalpy of MPUE without HEDS was 1.56 J g^−1^ for long-range ordered structure and 14.1 J g^−1^ for microcrystalline structure. At the BDO:HEDS ratio of 3:1, the melting enthalpy of (∆*H*_m2_) the microcrystalline structure was decreased to 9.5 J g^−1^, while the melting enthalpy of the long-range ordered structure was increased to 4.11 J g^−1^, due to mixed chain extenders increasing the structural asymmetry and reducing the crystallization of materials. When the BDO and HEDS molar ratio was 2:2, the melting peak of MPUE-SS-2 disappeared thoroughly. When only HEDS was added as the extender, the melting enthalpy of MPUE-SS-3 was 13.24 J g^−1^ for microcrystalline structure. The loss of melting peaks in microcrystalline hard segments demonstrated that the ordering of hard segments was severely hindered after adding mixed chain extenders instead of a single extender.

Dynamic mechanical properties are related to the thermal transition of microscale domains in polyurethane. The *T*_g_ value of MPUE and MPUE-SS was measured by tan δ peaks in DMA curves over a temperature range from −50 °C to 150 °C (Figure 9b). The *T*_g_ values of MPUE-SS-1, MPUE-SS-2, and MPUE-SS-3 were 13.2 °C, 38.3 °C, and 11.3 °C, respectively, which were significantly higher than that (−1.5 °C) of MPUE, suggesting that HEDS markedly enhanced the *T*_g_ of MPUE-SSs. The highest *T*_g_ value was found to be 38.3 °C when the molar ratio of HEDS to BDO was 2:2 for MPUE-SS-2. The sharp tan δ peak of MPUE-SS-2 was attributed to reduced crystallinity, which was consistent with the results of XRD (Figure 6) and DSC (Figure 8). These results demonstrated that the combined chain extenders could affect microphase separation and restrain the chain motion of soft segments.

### 3.3. Mechanical and Self-Healing Properties

All the MPUE and MPUE-SSs samples showed high tensile strength (Table 3 and Figure S1). The tensile strength of MPUE and MPUE-SS-1 was 30.0 MPa and 35.2 MPa, respectively (Table 3), suggesting that HEDS enhanced the mechanical properties of MPUE-SS. On the one hand, the disulfide bonds could be locked in the hard domains, and the disulfide exchange could be suppressed by constrained chain motion when the temperatures are below the glass transition temperatures (*T*_g_) of the hard microphase [20]. On the other hand, the tensile strength was improved by the smaller and uniformly dispersed hard domains playing a role of reinforcer and the enhanced compatibility of soft–hard phases due to the branched structure. With the increasing contents of HEDS, the tensile strength of MPUE-SSs was slightly decreased due to the disulfide exchange of disulfide bonds, which were dispersed in the soft domains [18,39].

To observe the self-healing performance of completely split MPUE-SSs, the dumbbell-shaped sample was cut in half and connected again, followed by self-healing at 120 °C for 24 h. As shown in Table 3, the self-healing efficiency was increased from 30.0% to 75.7% with increasing contents of HEDS. Without disulfide bonds, the self-healing efficiency of MPUE was about 30%, possibly due to hydrogen bonds between urethane groups. The self-healing performance of PUEs needs to be improved in spite of its transparency and high tensile strength. It has been found that a high content of hard segments is not conducive to the self-healing ability, because the constrained chain motion may suppress the disulfide exchange of disulfide bonds in hard domains [33]. Therefore, adjusting the contents of the hard phase is pivotal for taking advantage of urethane hydrogen and disulfide bonds. Here, we further explored the impacts of hard segment contents on the self-healing and mechanical properties of transparent PUEs, namely, MPUE-SS-4 and MPUE-SS-5. The Raman spectra (Figure S2), ^1^H-NMR (Figure S3), UV–visible transmittance spectra (Figure S4), XRD patterns (Figure S5), and dynamic thermomechanical analysis (DMA) curves (Figure S6) of the two samples are shown in the Supporting Information.

**Figure 10:**
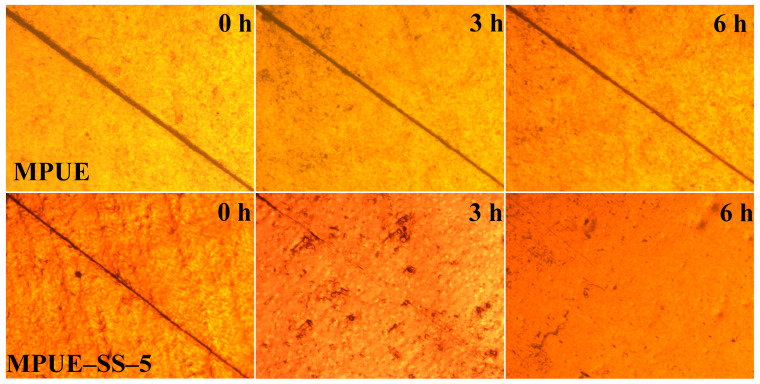
Representative images of MPUE and MPUE-SS-5 following heating at 120 °C for 3 and 6 h.

MPUE-SS-5 exhibited the largest maximum tensile strength of 39.9 MPa (Table 3 and Figure S1), which was much higher than those of previously reported self-healing materials [18,19,20,30,40]. The increasing contents of disulfide bonds could promote the uniform dispersion of hard segments in the soft phase, which could reinforce the physical crosslinking effects. Therefore, the tensile strength (39.9 MPa) of MPUE-SS-5 was much higher than that (27.4 MPa) of MPUE-SS-4. Moreover, the self-healing efficiency of MPUE-SS-5 improved significantly and reached 95%. The self-healing performance of MPUE-SS was also confirmed by microscopy observation (Figure 10). Here, MPUE-SS-5 was chosen to compare the self-healing performance with MPUE. The surface of each sample was cracked using a razor blade and heated at 120 °C. Clearly, the scratch of MPUE-SS-5 almost disappeared in about 6 h, while the scratch of MPUE remained.

### 3.4. Shape-Memory Performance

**Figure 11:**
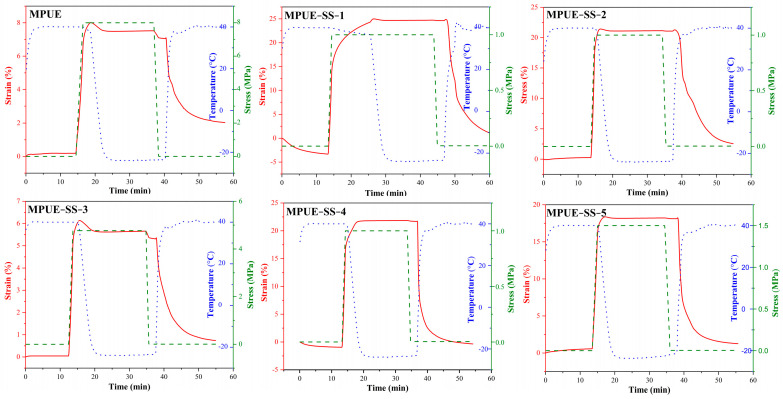
Shape-memory performance of MPUE and MPUE-SSs. Red solid lines, green dashed lines and blue dotted lines correspond to the strain, stress and temperature as a function of time, respectively.

In the thermal analyses (DSC and DMA) above, *T*_g_ was adopted as the phase transition temperature (*T*_trans_) to initiate the dual shape-memory effect. The dual shape-memory properties of MPUE and MPUE-SS are summarized in Table 3 and Figure 11. The architecture of HEDS significantly improved the shape-memory performance of MPUE-SSs. At the BDO:HEDS ratio of 1/1, the *R*_f_ and *R*_r_ of MPUE-SS-2 were 99.8% and 88.9%, respectively, which were higher than the 94.0% and 73.2% of MPUE. The addition of HEDS caused an increase in the *T*_g_ of PUEs, which resulted in the complete freezing of the segments in MPUE-SS-2 at −20 °C, thereby leading to increased *R*_f_. In addition, sparsely scattered hard domains, branching structure, and exchange of disulfide bonds can serve as physical network nodes to improve the *R*_r_.

It has been reported that there was critical fixed-phase content, at which the fixed phase can form a relatively complete physical crosslinked structure [41]. Therefore, we further investigated the impacts of different hard segment contents on shape-memory properties by effectively controlling the proportion and structure of the fixed and reversible phases. When the content of hard segments was less than 50%, the MPUE-SSs showed excellent shape-memory performance, with the *R*_f_ and *R*_r_ value reaching 99.6% and 96.0%, respectively (Table 4).

In MPUE-SSs, the glass transition of the soft segment serves as a reversible phase, while the sparsely scattered hard domains, branching structure, and disulfide exchange collectively act as the fixed phases. Above the *T*_g_ of the soft segment, PCL chains are also in a highly elastic state with flexible segments. By applying an external force, reversible deformation is achieved through the movement of PCL chains. Then, rapid cooling leads to the transition of the PCL chains into a glassy state, in which molecular chain movements are frozen to gain temporary shapes. Upon reheating, the PCL chains transition from a glassy state to a highly elastic state, leading to the release of tensile stresses. Owing to entropy elasticity, the PCL chains regain the initial shape. The increase in recovery rates is associated with an increase in crosslinking points. In a shape memory-assisted self-healing polyurethane system, the scratch was firstly healed by the shape-memory effects, followed by the disulfide exchange that accelerated the interdiffusion of polymer chains to establish the crosslinks on the fractured surfaces to achieve self-healing [42]. Therefore, excellent shape-memory effects can improve the self-healing performance.

**Scheme 2:**
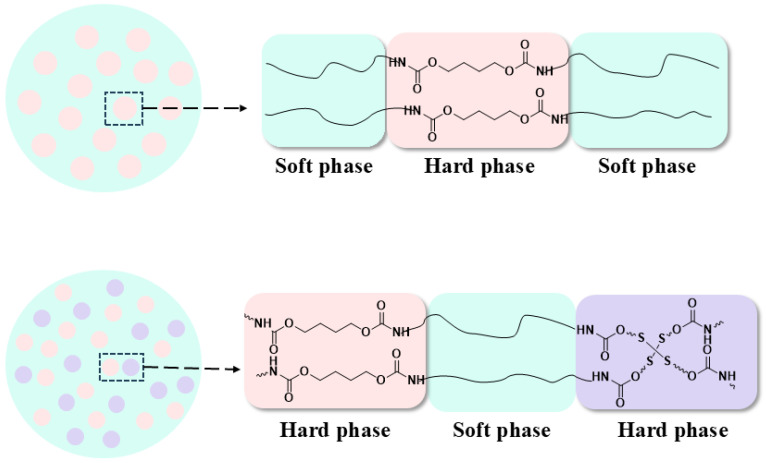
Illustration of the multiphase morphology of MPUE and MPUE-SSs.

Based on the above analysis, an interpretation of enhanced transparent, robust, shape-memory and self-healing properties of polyurethane elastomer via combining HEDS and BDO as the chain extenders was proposed. Firstly, the impact of combined BDO and HEDS extenders on the microphase morphology of MPUE-SS is schematically shown in Scheme 2. Compared with the MPUE using a single BDO extender, the combined chain extenders (HEDS and BDO) could lead to decreased hard domain size and uniform dispersion of hard domains in the soft phase. The smaller and uniformly dispersed hard domains play a role of reinforcer to enhance the tensile strength. The ordering of hard segments was severely hindered after adding combined chain extenders instead of a single extender, leading to the reduced crystallization of materials and high transparency. The decreased hard domain size and uniform dispersion of hard domains in the soft phase obtained via adjusting the molar ratio of HEDS to BDO act as the fixed phase via physical crosslinking effects, leading to the good shape-memory property. The disulfide bonds play a key role in the self-healing property enhancement of MPUE-SS. The reduced hard segment content via increasing the molar ratio of hydroxyl groups in diol to extender from 1:3 to 1:2 with a consistent molar ratio of HEDS to BDO could endow the soft segment with more disulfide bonds and further improve the self-healing property markedly.

## 4. Conclusions

In this work, a new transparent and self-healing PUE containing disulfide bonds and branched structure was synthesized. MDI-based PUE with disulfide bonds (MPUE-SSs) exhibited more robust mechanical properties and higher tensile strength compared with the previously reported self-healing polyurethane materials. MPUE-SSs possessed similar transparency to those of alicyclic polyurethane elastomers. Surface scratches were healed in 6 h, and half-cut MPUE-SSs reached a healing efficiency of over 95% in 24 h at 120 °C. Mechanical and self-healing properties were improved through rational molecular design (introducing aromatic isocyanates) and microphase control (phase-locking of dynamic bonds). In MPUE-SSs, viscoelastic hard domains can protect highly dynamic chemical bonds, which are easily locked and unlocked in response to temperature. The combined use of aliphatic disulfide compounds, BDO, and PPG-3 promoted the enhancement of transparency.

## Figures and Tables

**Table 1:** Details of the MPUE and MPUE-SSs with different ratios of BDO and HEDS.

Sample	n (OH Diol):n(NCO MDI):n(OH Extender)	BDO:HEDS	Sulfur Content ^a^ (wt%)	*M*_n_ (×10^4^)	*M*_w_ (×10^4^)	PDI ^b^	[η]	Hard Segment Content ^c^ (wt%)
MPUE	1:4:3	4:0	0	1.05	2.41	2.30	0.98	55.9
MPUE-SS-1	1:4:3	3:1	2.1%	1.09	2.68	2.46	1.01	57.7
MPUE-SS-2	1:4:3	2:2	4.1%	1.01	3.23	3.20	1.34	58.6
MPUE-SS-3	1:4:3	0:4	7.6%	0.98	2.13	2.17	0.74	60.3
MPUE-SS-4	1:2:1	2:2	2.0%	1.28	4.57	3.57	1.20	39.2
MPUE-SS-5	1:3:2	2:2	3.2%	3.27	12.59	3.85	1.63	50.8

^a^ The sulfur content of samples was calculated based on the molar feed ratio. ^b^ PDI, polydispersity index of polymer, is calculated via the ratio of *M*_w_ to *M*_n_. ^c^ The hard segment content of each sample was calculated based on the molar feed ratio, which was in accord with that calculated based on the peak integral area of the ^1^H-NMR spectrum.

**Table 2:** Parameters derived from DSC curves of MPUE and MPUE-SSs with different ratios of BDO and HEDS.

Sample	*T*_g, s_ (°C)	*T*_g, h_ (°C)	*T*_m1_ (°C)	∆*H*_m1_ (J g^−1^)	*T*_m2_ (°C)	∆*H*_m2_ (J g^−1^)	*T*_g_ (°C)
MPUE	−15.9	48.6	124.1	1.56	177	14.1	−1.5
MPUE-SS-1	−0.20	51.9	124.2	4.11	178	9.5	13.2
MPUE-SS-2	20.4	71.1	--- ^a^	---	---	---	38.3
MPUE-SS-3	8.2	---	---	---	172	13.24	11.3

^a^ --- means that no glass transition was detected.

**Table 3:** Mechanical properties of MPUE and MPUE-SSs with different ratios of BDO and HEDS.

Samples	Original		After 24 h Healing		Self-Healing Efficiency (%)
	Tensile Strength (MPa)	Elongation at Break (%)	Tensile Strength (MPa)	Elongation at Break (%)	
MPUE	30.0 ± 0.5	380 ± 5	9.0 ± 0.3	60.8 ± 3	30.0 ± 0.5
MPUE-SS-1	35.2 ± 0.2	273 ± 2	18.7 ± 0.3	66.2 ± 6	53.2 ± 1.2
MPUE-SS-2	33.9 ± 0.2	292 ± 3	23.3 ± 0.2	194 ± 2	68.7 ± 0.6
MPUE-SS-3	27.2 ± 0.3	292 ± 8	20.6 ± 0.5	31.2 ± 1	75.7 ± 1.3
MPUE-SS-4	27.4 ± 0.5	453 ± 9	16.6 ± 0.3	525 ± 10	65.3 ± 0.9
MPUE-SS-5	39.9 ± 0.3	285 ± 2	36.9 ± 0.5	308 ± 1	95.0 ± 2.8

**Table 4:** Shape-memory performance of MPUE and MPUE-SSs.

Samples	*R*_f_ (%)	*R*_r_ (%)
MPUE	94.0	73.2
MPUE-SS-1	99.8	85.0
MPUE-SS-2	99.8	88.9
MPUE-SS-3	94.0	86.7
MPUE-SS-4	99.4	96.0
MPUE-SS-5	99.6	96.0

## Data Availability

The data presented in this study are available on request.

## Supplementary Materials

The following supporting information can be downloaded at: https://www.mdpi.com/article/10.3390/polym17091243/s1, Figure S1: Stress–strain curves for MPUE and MPUE-SS before and after healing at 120 °C for 24 h; Figure S2: Raman spectra of MPUE-SS with different hard segment contents; Figure S3: ^1^H-NMR of MPUE-SS with different hard segment contents; Figure S4: UV–visible transmittance spectra of MPUE-SS with different hard segment contents; Figure S5: XRD patterns of MPUE-SS with different hard segment contents; Figure S6: DMA curves of MPUE-SS with different hard segment contents; Table S1: The integral area of peaks and calculated hard segment content of each sample.
