# Supplementary material for: Construction of a Transparent, Robust, Shape-Memory and Self-Healing MDI-Based Polyurethane Elastomer
Source: Polymers (Basel). 2025 May 2;17(9):1243. doi: 10.3390/polym17091243 (PMC12073319; doi:10.3390/polym17091243)
Supplement: Supplementary file 1 [file polymers-17-01243-s001.zip › polymers-3588301-supplementary.pdf]

Supporting Information

Construction of a transparent, robust, shape-memory and self-healing MDI-based polyurethane elastomer

Haichun Dang[1], Ziliang Zhang[1], Ruibing Sun[1], Yunlun Li[1], Mengyu Lin[1], Siting Yang[1], Maoyong He[1], Zhaozan Xu[2,], Xiangcheng Bian[1,] **

1 Department of Materials Engineering, Taiyuan Institute of Technology, Taiyuan 030008, China;

2 Institute of Resources and Environmental Engineering, Shanxi University, Taiyuan 030006, China

* Corresponding author email: zhaozanxu@sxu.edu.cn (Zhaozan Xu);

bianxiangcheng@126.com (Xiangcheng Bian)

Characterizations

The calculus of the disulfide content and of the MPUE and MPUE-SSs samples:

$$S = \frac{2M_s}{M_{PPG-3} \cdot n_{PPG-3} + M_{PCL} \cdot n_{PCL} + M_{MDI} \cdot n_{MDI} + M_{BDO} \cdot n_{BDO} + M_{HEDS} \cdot n_{HEDS}} \times 100\%$$

$$HS = \frac{M_{MDI} \cdot n_{MDI} + M_{BDO} \cdot n_{BDO} + M_{HEDS} \cdot n_{HEDS}}{M_{PPG-3} \cdot n_{PPG-3} + M_{PCL} \cdot n_{PCL} + M_{MDI} \cdot n_{MDI} + M_{BDO} \cdot n_{BDO} + M_{HEDS} \cdot n_{HEDS}} \times 100\%$$

where disulfide content and hard segment content denotes the disulfide and hard phase content of the samples, %, respectively; M_S denotes the relative atomic mass of the Sulphur atom; M_{PPG-3}, M_{PCL}, M_{MDI}, M_{BDO} and M_{HEDS} donate the mass fraction of PPG-3, PCL, MDI, BDO and HEDS, respectively; n_{PPG-3}, n_{PCL}, n_{MDI}, n_{BDO} and n_{HEDS} donate the molar ratio of the PCL and the HEDS in the system.

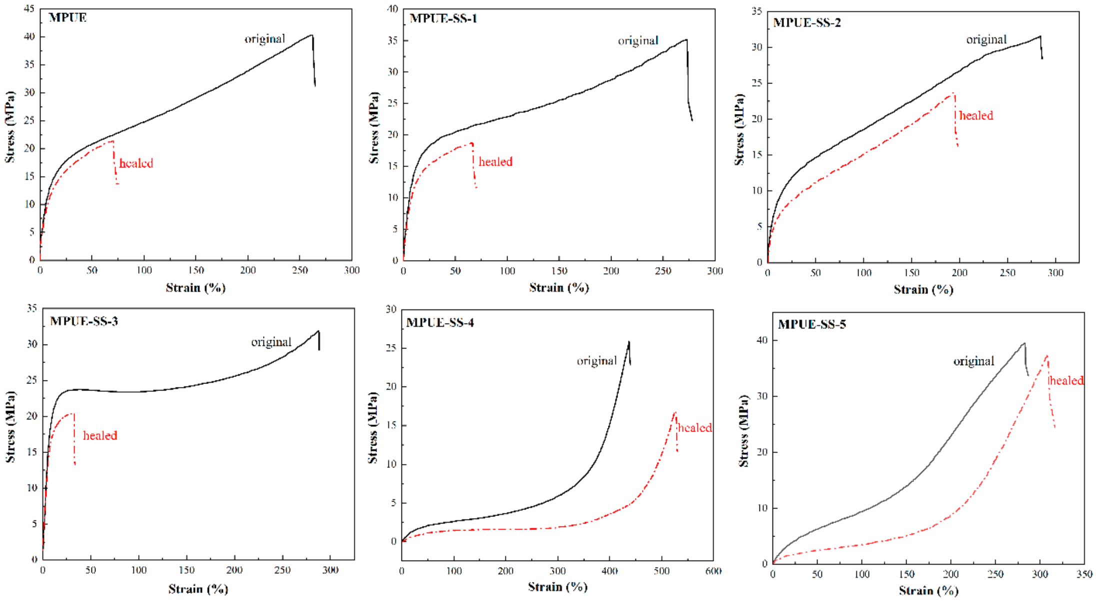

Figure S1. Stress-strain curves for MPUE and MPUE-SS before and after healing at 120 °C for 24 h

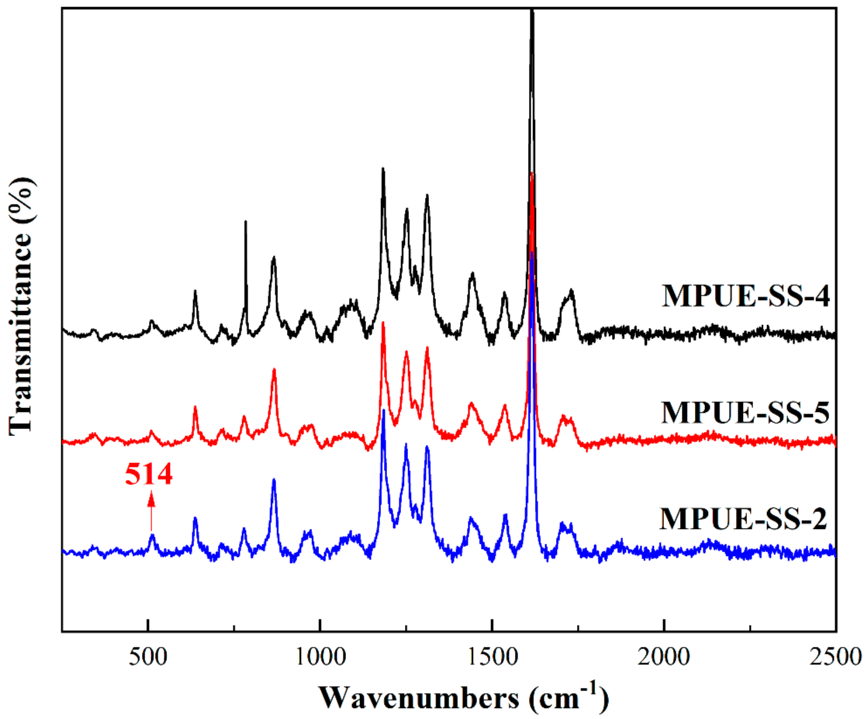

Figure S2. Raman spectra of MPUE-SS with different hard segment contents

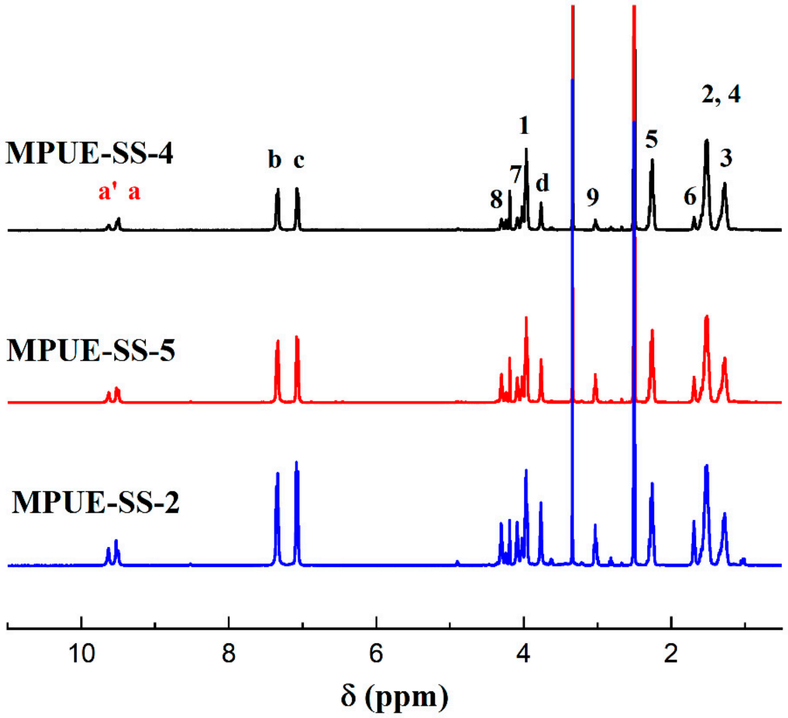

Figure S3. ^{1}H-NMR of MPUE-SS with different hard segment contents

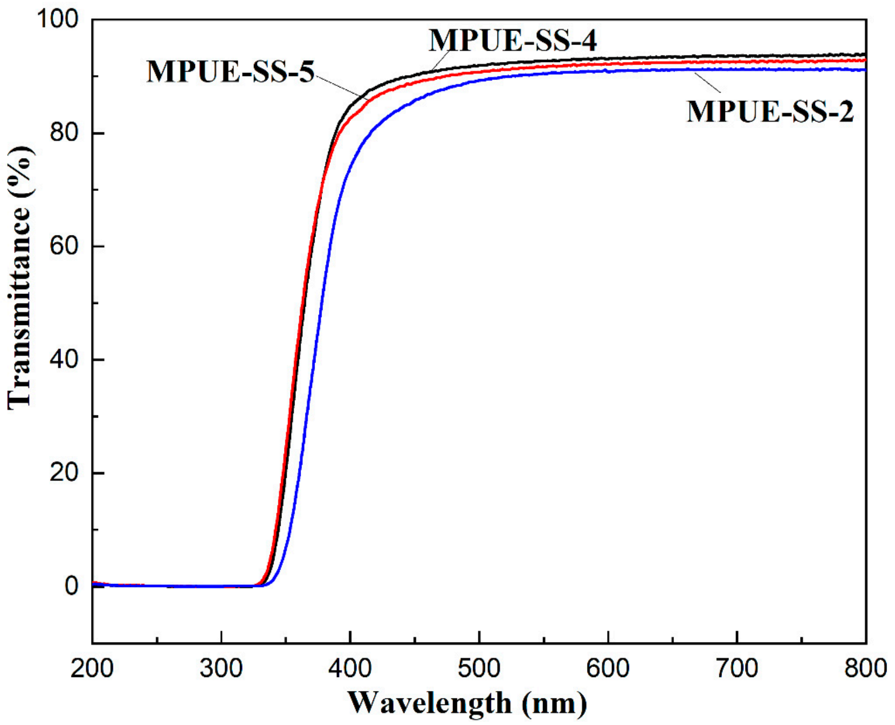

Figure S4. UV-visible transmittance spectra of MPUE-SS with different hard segment contents

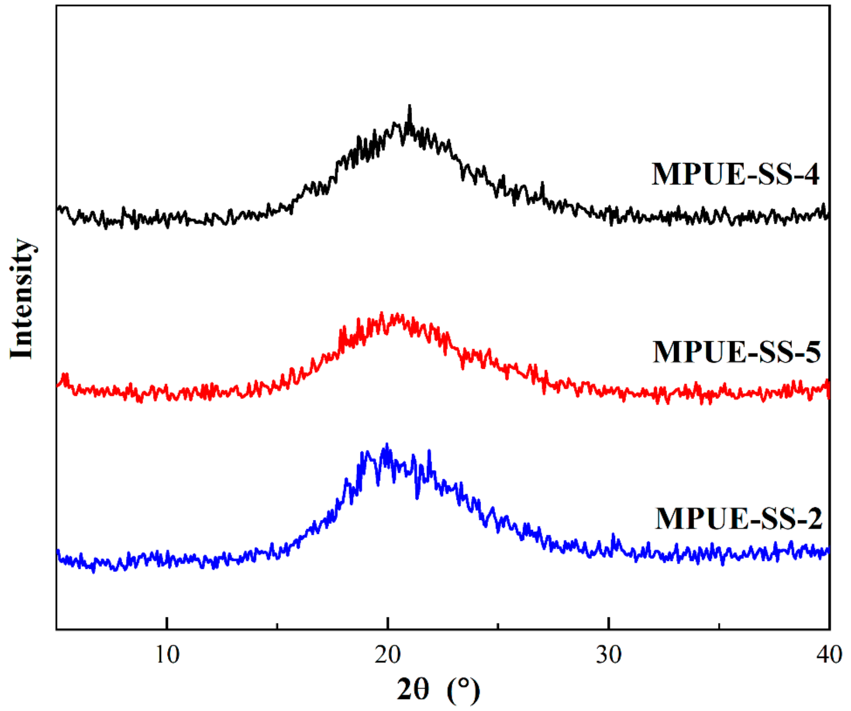

Figure S5. XRD patterns of MPUE-SS with different hard segment contents

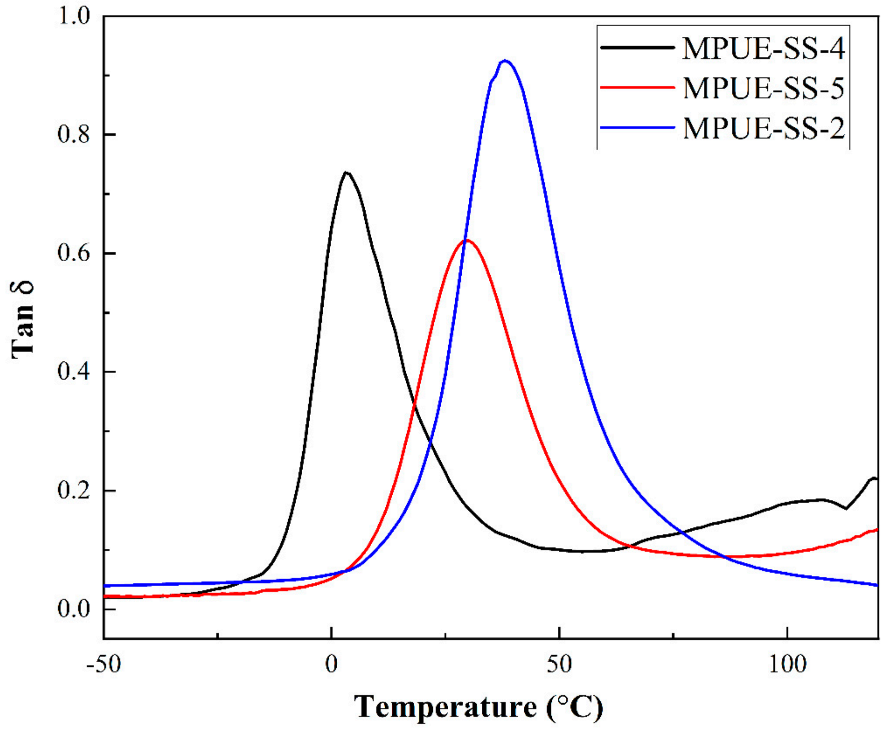

Figure S6. DMA curves of MPUE-SS with different hard segment contents

Table S1 The peak integral area of ^{1}H-NMR spectrum and calculated hard segment content of each sample

Sample	Integral area of peaks						Hard segment content[a] (wt %)
	H-a,a' (N-H)	H-b (MDI)	H-9 (HEDS)	H-6 (BDO)	H-3 (PCL)	H-0 (-CH_3 of PPG-3)	
MPUE	1.00	2.00	0	1.44	2.01	0.13	54.8
MPUE-SS-1	1.00	2.00	0.34	1.11	1.99	0.07	57.9
MPUE-SS-2	1.00	2.01	0.68	0.78	2.02	0.21	60.8
MPUE-SS-3	1.00	1.98	1.35	0	2.04	0.08	63.4
MPUE-SS-4	1.00	1.94	0.47	0.56	3.80	0.14	41.4
MPUE-SS-5	1.00	1.98	0.63	0.67	2.42	0.11	54.4

[a] The hard segments of samples were calculated based on the peak integral area of ^{1}H-NMR spectrum.
